# Supplementary material for: Calcineurin-inhibitor free immunosuppression after lung transplantation – a single center case-control study in 51 patients converted to Mechanistic Target of Rapamycin (mTOR) inhibitors
Source: PLoS One. 2023 May 18;18(5):e0284653. doi: 10.1371/journal.pone.0284653 (PMC10194991; doi:10.1371/journal.pone.0284653)
Supplement: S1 Table — (DOCX) [file pone.0284653.s001.docx]

|  | Patients with malignancy and CNI-free immunosuppression n=37 | Patients with malignancy and continued CNI-medication, n=165 | p |
| --- | --- | --- | --- |
| Gender, female, n (%) | 14 (38) | 66 (40) | 0.808 |
| Age at transplant (years), median (25^th^, 75^th^ percentile) | 48 (41, 57) | 52 (36, 58) | 0.683 |
| Age at malignancy diagnosis (years), median (25^th^, 75^th^ percentile) | 58 (50, 63) | 57 (47, 64) | 0.831 |
| Transplant procedure, n (%) |  |  | 0.756 |
| Bilateral | 30 (81) | 136 (82) |  |
| Single lung | 5 (14) | 21 (13) |  |
| Heart-lung | 2 (5) | 5 (3) |  |
| Lung-liver | - | 3 (2) |  |
| Diagnosis, n (%) |  |  | 0.386 |
| COPD / alpha1ATD | 10 (27) | 54 (33) |  |
| Fibrosis / interstitial lung disease | 12 (32) | 53 (32) |  |
| CF / bronchiectasis | 9 (24) | 32 (19) |  |
| PH / vascular diseases | 4 (11) | 7 (4) |  |
| Other | 2 (5) | 19 (12) |  |
| Malignancy diagnosis after transplant (months), median (25^th^, 75^th^ percentile) | 74 (30, 125) | 59 (21, 102) | 0.384 |
| Approach, n (%) |  |  | 0.020 |
| Curative approach | 11 (30) | 84 (51) |  |
| Palliative approach | 26 (70) | 81 (49) |  |
| CLAD, n (%) | 20 (54) | 75 (46) | 0.360 |
| Date onset before malignancy | 15 (41) | 53 (32) | 0.327 |
| Date onset after malignancy | 5 (14) | 22 (13) | 1.000 |
| ReTx after malignancy, n (%) | - | 7 (4) | 0.354 |
| Deceased, n (%) | 31 (84) | 108 (65) | 0.030 |
| Cause of death, n (%) |  |  | 0.737 |
| Unknown | 2 (6) | 8 (7) |  |
| CLAD | 5 (16) | 18 (16) |  |
| Infection | 1 (3) | 12 (11) |  |
| Malignancy | 21 (68) | 62 (56) |  |
| Cardiovascular | 1 (3) | 3 (3) |  |
| Other | 1 (3) | 8 (7) |  |
| Post-transplant survival (months), median (95%-CI) | 113 (55, 171) | 108 (95, 121) | 0.555 |
| Post-malignancy survival (months), median (95%-CI) | 20 (10, 30) | 19 (9, 29) | 0.414 |
| Post-transplant follow-up (months), median (25^th^, 75^th^ percentile) | 97 (42, 147) | 92 (48, 148) | 0.626 |
| Post-malignancy follow-up (months), median (25^th^, 75^th^ percentile) | 20 (9, 41) | 13 (3, 42) | 0.148 |

**S1 Table: Characteristics of lung transplant recipients with malignancy**

CLAD – chronic lung allograft dysfunction, CNI – calcineurin inhibitors
