# Supplementary material for: Calcineurin-inhibitor free immunosuppression after lung transplantation – a single center case-control study in 51 patients converted to Mechanistic Target of Rapamycin (mTOR) inhibitors
Source: PLoS One. 2023 May 18;18(5):e0284653. doi: 10.1371/journal.pone.0284653 (PMC10194991; doi:10.1371/journal.pone.0284653)
Supplement: Attachment — Submitted filename: Reviewer_P2P response PLOSone LTx_CNIfree07022023.docx [file pone.0284653.s003.docx]

| **PONE-D-22-28204: Title: “Calcineurin-Inhibitor Free Immunosuppression After Lung Transplantation – a Single Center Case-control Study in 51 Patients converted to Mechanistic Target of Rapamycin (mTOR) Inhibitors"**  **EMID:9ed5341f17931db9** | | |
| --- | --- | --- |
| *Page and line numbers refer to the marked version of the revised version of the manuscript | | |
| Editorial Office | | |
| # | Requirements and prompts | Response* |
| 1 | Please ensure that your manuscript meets PLOS ONE's style requirements, including those for file naming. The PLOS ONE style templates can be found at <https://journals.plos.org/plosone/s/file?id=wjVg/PLOSOne_formatting_sample_main_body.pdf>  and  <https://journals.plos.org/plosone/s/file?id=ba62/PLOSOne_formatting_sample_title_authors_affiliations.pdf> | File names were renamed according to PLOS One standards. Headings and Tables were reformatted according to PLOS ONE guidelines. Author´s names affiliations were adapted according to PLOS One standards. Tables were incorporated in text body in the paragraph following first mentioning as requested. |
| 2 | Please provide additional details regarding participant consent. In the ethics statement in the Methods and online submission information, please ensure that you have specified what type you obtained (for instance, written or verbal, and if verbal, how it was documented and witnessed). If your study included minors, state whether you obtained consent from parents or guardians. If the need for consent was waived by the ethics committee, please include this information | This information was added to the Methods section of the revised version of the manuscript (p 6, line 119) |
| 3 | We note that you have indicated that data from this study are available upon request. PLOS only allows data to be available upon request if there are legal or ethical restrictions on sharing data publicly. For more information on unacceptable data access restrictions, please see <http://journals.plos.org/plosone/s/data-availability#loc-unacceptable-data-access-restrictions>. | Data availability was adapted in the Materials and Methods section of the revised version of the manuscript (p 21/22, lines 343-347) |
| a | If there are ethical or legal restrictions on sharing a de-identified data set, please explain them in detail (e.g., data contain potentially sensitive information, data are owned by a third-party organization, etc.) and who has imposed them (e.g., an ethics committee). Please also provide contact information for a data access committee, ethics committee, or other institutional body to which data requests may be sent. | Not applicable |
| b | If there are no restrictions, please upload the minimal anonymized data set necessary to replicate your study findings as either Supporting Information files or to a stable, public repository and provide us with the relevant URLs, DOIs, or accession numbers. For a list of acceptable repositories, please see <http://journals.plos.org/plosone/s/data-availability#loc-recommended-repositories>.We will update your Data Availability statement on your behalf to reflect the information you provide. | A minimal data set was uploaded during submission. |
| PACE | While revising your submission, please upload your figure files to the Preflight Analysis and Conversion Engine (PACE) digital diagnostic tool, <https://pacev2.apexcovantage.com/>. PACE helps ensure that figures meet PLOS requirements. To use PACE, you must first register as a user. Registration is free. Then, login and navigate to the UPLOAD tab, where you will find detailed instructions on how to use the tool. If you encounter any issues or have any questions when using PACE, please email PLOS at [figures@plos.org](mailto:figures@plos.org). Please note that Supporting Information files do not need this step. | All Figures were uploaded to Preflight Analysis and Conversion Engine (PACE) and the uploaded files |
| Reviewer 1 | | |
| # | Reviewer Comment | Response* |
| 1 | This is a retrospective analysis rather than prospective study and the mTOR inhibitor was started at varying intervals for the treatment group. | The reviewer is correct and this information was included in the limitation section in the discussion (p11 line xxx) |
| 2 | The group of patients with malignancies is very small (51, compared to 2049). | The reviewer is right. The small number of malignant disease converted to CNI-free immunosuppression highlights the rarity of this approach in clinical routine. No change was added to the revised version. |
| 3 | Multiple types of cancer are being compared together | We agree. We added this information to the limitation section in the discussion (p19 line 298-302). |
| 4 | The CNI free immunosuppression was initiated after the diagnosis of malignancy was made, thus not allowing for an analysis of the "preventive" potential of mTOR inhibitors used as CNI free immunosuppression. | The reviewer is correct and we added this information to the limitation section in the discussion (p19 line 298-302) |
| 5 | mTOR inhibitors have been used in Lung transplant programs for many years, although this reviewer acknowledges that they are seldom used in an elective or protocolized fashion. Effectiveness of immunosuppression is well established, so there is nothing novel in reporting that they are well tolerated. Unfortunately, this study does not help to answer the malignancy question as the immunosuppression was changed "after the fact". A better approach would be to develop a protocolized conversion timeline, with regular sequential follow up. | We agree with the reviewer and we added this suggestion to the outlook section in the discussion of the revised version of the manuscript (p19 line 298-302) |
| Reviewer 2 | | |
| # | Reviewer Comment | Response* |
| 1 | I was delighted to have a chance to peer review the submission to PLOS ONE entitled “Calcineurin-Inhibition Free Immunosuppression After Lung Transplantation – a Single Center Case-Control Study in 51 Patients converted to Mammalian Target of Rapamycin (mTOR)”, submitted by the Hannover group. In it, you report on a retrospective analysis of 51 patients over almost 30 years at a single center where patients had their CNI stopped and an alterative regimen was used. Your aim was to “investigate indications, safety, and outcomes of CNI-free immunosuppression based on mTOR inhibitors in the cohort” (last sentence of the introduction). About half of them were patients who had been changed because of active malignancies. Some later had CNI’s re-introduced. | Thank you for appreciating our work and nice summary. |
| 2-1 | You describe better than expected outcomes (in my estimation), with no acute rejections reported (but limited numbers having had biopsies), high mortality for the oncology patients (1-year survival of 36%), low mortality for the non-oncology patients (1-year survival 100%), improvement in neurologic side effects for those who had been changed for that reason, and a modest improvement in GFR for the cohort. The median duration of CNI-free immunosuppression was 338 days, with the period beginning a median of 6.1 years after transplantation.  1. Why were there not more biopsies done after the switch? | . Surveillance transbronchial biopsy after conversion to a new maintenance regimen was performed since 2018. This information is included in the Materials and Methods section (page 7, line 115-118). |
| 2-2 | 2. Other than “CLAD or not”, were there any assessments of graft health after the switch? What happened to the spirometry? | Spirometry was part of our regular screening during personal visits. Any change in FEV1 of more than 10% from previous value led to prompt investigations for work up including bronchoscopy. The same approach was applied for new hypoxemia or new opacities on imaging. This information is included in the Materials and Methods section (page 7, line 120-123). |
| 2-3 | 3. You state in the results section that only 47% of the cohort of 51 patients had been biopsied at any time before the CNI free conversion. This is surprising to me and may need clarification. Are not all of your center’s patients getting transbronchial biopsies in their first year after transplant? If not, you may want to mention somewhere that this is your routine. | We started our surveillance biopsy program after 2009. Patients transplanted before 2009 did not have regular protocol biopsies |
| 2-4 | You state that 10 patients tested positive for donor specific antibodies before conversion. Please mention how you handled this information in general (did it influence decisions about converting to CNI-free?). Were patients routinely (or occasionally ad hoc) testing for DSAs after the conversion? | Screening for donor-specific antibodies was performed since 2018 during the first postoperative year. Routine testing after conversion was not performed. Presence of donor-specific antibodies was not a contraindication for conversion. This information is included in the Materials and Methods section (page 7, line 115-118). |
| 2-5 | Your figures should each have a text box for the document with a description of what is in the figure. | Title of the figures and tables are listed on page 19 lines 320-330 in the revised version of the mansucript |
| 2-6 | It would be interesting and visually pleasing to see survival curves (ie Kaplan-Meier) | We are grateful for this valuable comment. Our oncological statistical review advised against the use of Kaplan-Meier curves due to varying intervals of starting mTORinhibitors in treatment group and control group. Nevertheless, a KM curve with survival after conversion was constructed during drafting of the manuscript. It is displayed here:  .  Because of statistical concerns we still decided against the publication of this graph. |
| 2-7 | Under “2.2 Safety”, you describe incidence of hospitalization and infections in the group that was CNI free. How did this compare to patients who were on more standard therapy? You might consider a broader comparison looking at all patients (not just oncology patients) and outcomes with and without the conversion to CNI-free. | Thanks for this comment (see comment also 2-7). Due to varying intervals of starting mTORinhibitors in the treatment group and control group number of hospitalisations is very difficult to compare. It is well known that cancer patients with a non-curative approach have a high risk of hospitalisation after diagnosis. Since these patients represent the majority of CNI-free patients in our study and small numbers we decided against an additional analysis. |
| Minor 1 | In the first paragraph of the introduction, the word “haltering” was used. This is an irregular term and I might suggest using a different word such as “slowing”. | Changed on p 4 line 66 |
| Minor 2 | In the last sentence of the first paragraph of the introduction, change “CNI-free immunosuppression after lung transplantation is limited” to “CNI-free immunosuppression after lung transplantation are limited”. | Changed on p 4 line 69 |
| Minor 3 | Throughout the document, mTOR is referred to as “mammalian target of Rapamycin”. A more updated and common terminology is now “mechanistic target of Rapamycin”. Consider a change if you are inclined to agree. | The reviewer is right. The MeSH term “mechanistic target of Rapamycin” was introduced in 2018. This was changed throughout the manuscript (including title) |
| Minor 4 | The word “intermitted” just before section “2.1 outcomes” is an irregular word, please change. | Intermitted was changed to intermittent on page 13, line 190 |
| Minor 5 | In the “2.1 Outcomes” section, consider changing the description of “corona virus disease 2019” to “SARS CoV-2 infection”. | Severe acute respiratory syndrome coronavirus type 2 infection was used instead on page 13 (line 196) and 16 (line 231) |
| Minor 6 | In Discussion, “Ina conference abstract Eiting” should be, “In a conference abstract Eiting” | Done. |
| Minor 7 | Discussion, “In our cohort, Minor improvement” should be, “In our cohort, minor improvement” | Done. |
