# Supplementary material for: Calcineurin-inhibitor free immunosuppression after lung transplantation – a single center case-control study in 51 patients converted to Mechanistic Target of Rapamycin (mTOR) inhibitors
Source: PLoS One. 2023 May 18;18(5):e0284653. doi: 10.1371/journal.pone.0284653 (PMC10194991; doi:10.1371/journal.pone.0284653)
Supplement: Attachment — Submitted filename: Reviewer_P2P response PLOSone LTx_CNIfree20032023.docx [file pone.0284653.s004.docx]

| **PONE-D-22-28204R1: Title: “Calcineurin-Inhibitor Free Immunosuppression After Lung Transplantation – a Single Center Case-control Study in 51 Patients converted to Mechanistic Target of Rapamycin (mTOR) Inhibitors"**  **EMID:9ed5341f17931db9** | | |
| --- | --- | --- |
| *Page and line numbers refer to the marked version of the revised version of the manuscript | | |
| Editorial Office | | |
| # | Requirements and prompts | Response* |
| 1 | A rebuttal letter that responds to each point raised by the academic editor and reviewer(s). You should upload this letter as a separate file labeled 'Response to Reviewers'. | A detailed Point-to-point response was added to the re-submission of the revised version of the manuscript. |
| 2 | A marked-up copy of your manuscript that highlights changes made to the original version. You should upload this as a separate file labeled 'Revised Manuscript with Track Changes'. | A marked version of the revised version with tracked changes was uploaded.  Label 'Revised Manuscript with Track Changes'. |
| 3 | An unmarked version of your revised paper without tracked changes. You should upload this as a separate file labeled 'Manuscript'. | A unmarked version of the revised version without tracked changes was uploaded.  Label 'Manuscript' |
| Reviewer 2 | | |
| # | Reviewer Comment | Response* |
| 1 | Thank you for your work in amending your submission. It is improved with your address of reviewers' comments. | Thank you for the compliment. |
| Reviewer 3 | | |
| # | Reviewer Comment | Response* |
| 1 | I reveiwed a revised version of the manuscript entitled "Calcineurin-Inhibitor Free Immunosuppression After Lung Transplantation – a Single Center Case-control Study in 51 Patients converted to Mechanistic Target of Rapamycin (mTOR) Inhibitors" by Gttlieb et al.  First of all I have to state that I did NOT review the previous version.  Due to the nature of the study (retrospective single-center study) it is difficult to draw any conclusion. | Thank you for clarifying this. For authors it is very difficult to deal with new reviewers without having knowledge of previous track changes. We´ll do our best to comply. |
| 2 | For those patients who were converted from CNI-based IS to mTOR inhibitor-based IS the trough levels of sirolimus and everolimus are not mentioned. | Target trough levels are mentioned in the Materials and Methods section of the revised version of the manuscript on page 4 line 88. With more experience with mTOR based CNI-free immunosuppression we lowered our target trough levels to 8-12 ng/ml in recent years |
| 3 | On average after conversion from CNI- to mTOR-I-based IS how many times the patients were seen in the outpatient clinic | Patient were postoperatively seen in 4-6 weeks interval in the first year, every three months in the second year, every six months up to year 5 and yearly thereafter Patients with new medical problems were usually seen in monthly or 3-monthly intervals. This information is included in the Materials and Methods section of the revised version of the manuscript (page 4, line 106-107). |
| 4 | mTOR-Is-associated side effects are poorly reported: when it occurred did it result in reverse conversion to CNI-based IS? | Side effects of mTOR-IS are reported the results section of the revised version of the manuscript (page 13, line 226-230). 11 patients were reconverted to CNI free immunosuppression due to side effects; 4 patients due to other reasons. Most frequent side effects leading to termination of CNI-free IS were GI complaints (diarrhea, mucositis, together 66%). 21 patients were hospitalized due to infections on mTOR-IS and in a single patient mTOR-IS was terminated due to recurrent infection |
| 5 | The paper is more suited for a pulmonology journal. Otherwise it could be considered as a Letter to the Editor. | According to previous editorial and reviewer comments the paper tracked further as an original publication. |
| Reviewer 4 | | |
| # | Reviewer Comment | Response* |
| 1 | Gottlieb and coll described their experience of CNI-free IS in lung transplant recipients.  In my opinion, the manuscript should be reoriented toward the main result of the study, ie the safety regarding regarding rejection, in lung transplant recipients with an advanced cancer. | We agree that safety information should be more strengthened and elaborated the results on biopsy results in lines 238-242 in the results section of the revised version of the manuscript on pages 13/14. |
| 2 | In a methodological point of vue, I think the comparison with patients under CNI that presented different cancer types, and different cancer stages prevents analysis of patient survival grouped by IS | We agree with this comment and this information is contained under limitations page 17, line 296-297. |
| 3 minor | introduction: the statement that CNI free IS is a reality in the kidney and heart or pancreas transplant setting should be tempered. In fact all of these CNIfree protocols were stopped and antimetabolite are switched to everolimus in association with CNI. | The statement was tempered (introduction, page 3, line 63) |
| 4 minor | A larger presentation of the burden of cancer after lung transplantation, and specificities of cancer in the lung transplantation could improve the intro. | Informations about the burden of cancer was included on page 3. Lines 75-77. |
| 5 minor | Patients and Method  The authors stated that they used only nonparametric tests for quantitative comparisons, so with different different distribution in a small cohort. This suggest that cox-regression model to assess predictive factors is not really useful in this case-series. I suggest to remove this part, or at least to develop the statistical section to explain the number of variables included in the model, and the number of events per variables | The requested information including the variables of interest of the cox regression model are listed in the stastitics section page 7, lines 145-147. |
| 6 minor | results: treatments used for cancer in association with mTOR should be developed to better understand the risk for rejection in this cohort | Chemotherapy was used in the majority of cancer patients (n=23), radiation was used in 12 and 16 patients underwent surgery for surgery. This information was added to the results section (page 9, lines 171-173) |
